# Coronavirus disease 2019-associated rapidly progressive organizing pneumonia with fibrotic feature: Two case reports

**DOI:** 10.1097/MD.0000000000021804

**Published:** 2020-08-28

**Authors:** Satoshi Okamori, Ho Lee, Yasushi Kondo, Yuto Akiyama, Hiroki Kabata, Yuko Kaneko, Makoto Ishii, Naoki Hasegawa, Koichi Fukunaga

**Affiliations:** a Division of Pulmonary Medicine; b Division of Rheumatology, Department of Medicine; c Center for Infection Diseases and Infection Control, Keio University School of Medicine, Shinjuku, Tokyo, Japan.

**Keywords:** coronavirus disease 2019, organizing pneumonia, corticosteroids

## Abstract

**Introduction:**

Pneumonia is one of the most important characteristics of coronavirus disease 2019 (COVID-19) and imaging findings of COVID-19 pneumonia are diverse and change over disease course. However, the detailed clinical course of organizing pneumonia (OP) caused by COVID-19 has not been clarified.

**Patient concerns:**

A 60-year-old man and a 61-year-old woman diagnosed with mild COVID-19 were admitted to our hospital. Their respiratory symptoms were deteriorating even after initiating treatment with antiviral drugs.

**Diagnosis:**

Chest X-rays and computed tomography scan showed a rapid progression of linear consolidation with reversed halo sign, distributed in subpleural and peri-bronchial regions. They also presented with pulmonary fibrosis findings, including traction bronchiectasis and marked lung volume reduction. They were diagnosed with rapidly progressing OP.

**Interventions:**

They were treated with systemic corticosteroids.

**Outcomes:**

The patients’ imaging findings and respiratory conditions improved rapidly without any adverse effects.

**Conclusion:**

Physicians should carefully monitor patients with COVID-19, as they can develop rapidly progressive and fibrotic OP, which respond to corticosteroids.

## 1 Introduction

The novel coronavirus disease 2019 (COVID-19) is spreading worldwide. Although pneumonia is 1 of the most important clinical characteristics of the disease,^[1]^ the detailed features and its appropriate treatment are not yet clear. Chest computed tomography (CT) is a useful diagnostic tool for COVID-19. Although multifocal patchy ground-glass opacity (GGO) and consolidations are common, the CT findings of COVID-19 pneumonia are diverse and change over disease course.^[2]^ Some patients have been reported to have findings suggestive of organizing pneumonia (OP) such as consolidation or reversed halo sign.^[2]^ However, there are few case reports detailing the clinical and treatment course of OP caused by COVID-19.

Herein, we describe 2 cases of COVID-19-associated OP. Notably, both patients had rapid reduction in lung volume and respiratory failure accompanied by findings of pulmonary fibrosis on chest CT. Antiviral drugs had no apparent effect on disease progression, and both cases were successfully treated with systemic corticosteroid therapy.

## 2 Case presentation

### 2.1 Case 1

**Figure 1:**
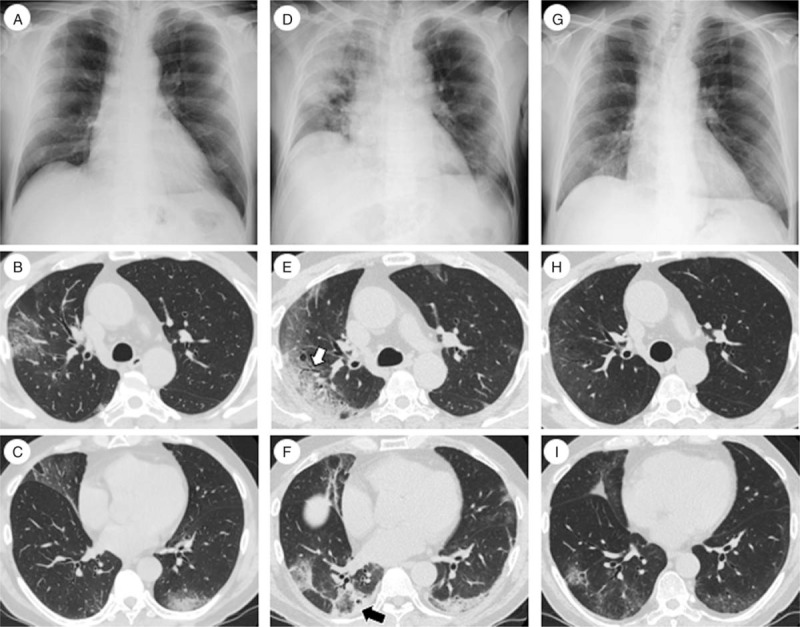
Chest imaging of Case 1. (A) Chest X-ray on admission. A slight ground-glass opacity (GGO) was observed in the right lung. (B, C) Chest computed tomography (CT) on admission revealed bilateral patchy GGO that was predominantly distributed throughout subpleural areas. (D) Chest X-ray on day 6 post hospital admission (taken immediately before corticosteroid administration) revealed consolidations and marked lung volume loss. (E, F) Chest CT on day 6. Consolidations developed bilaterally, some with band-like shapes. A central GGO surrounded by ring-shaped areas of consolidation (reversed halo sign) was observed (black arrow). Some GGOs were accompanied by traction bronchiectasis (white arrow). The volume of both lower lobes was decreased. (G) Chest X-ray and (H, I) Chest CT on day 23 revealed improvement of lung infiltration and recovery of lung volume. CT = computed tomography.

A 60-year-old man presented with a 7-day history of fever and cough. He had a medical history of gastroesophageal reflux disease and dyslipidemia. On admission, he presented with a pulse of 85 bpm, temperature of 38.5 °C, blood pressure of 132/88 mmHg, and oxygen saturation of 96% when breathing air. Laboratory data revealed elevated serum LDH (351 U/L), AST (149 U/L), ALT (82 U/L), and C-reactive protein (CRP) (8.23 mg/dL). Lymphopenia and coagulopathy were not observed. Chest X-ray and CT scan revealed patchy GGO predominantly distributed in the subpleural regions of the lungs (Fig. 1A–C). Real-time reverse transcription polymerase chain reaction (RT-PCR)-based tests for severe acute respiratory syndrome (SARS) coronavirus 2 (SARS-CoV-2) virus were positive. The patient was diagnosed with COVID-19 pneumonia and treated with levofloxacin, ciclesonide, and favipiravir.

**Figure 2:**
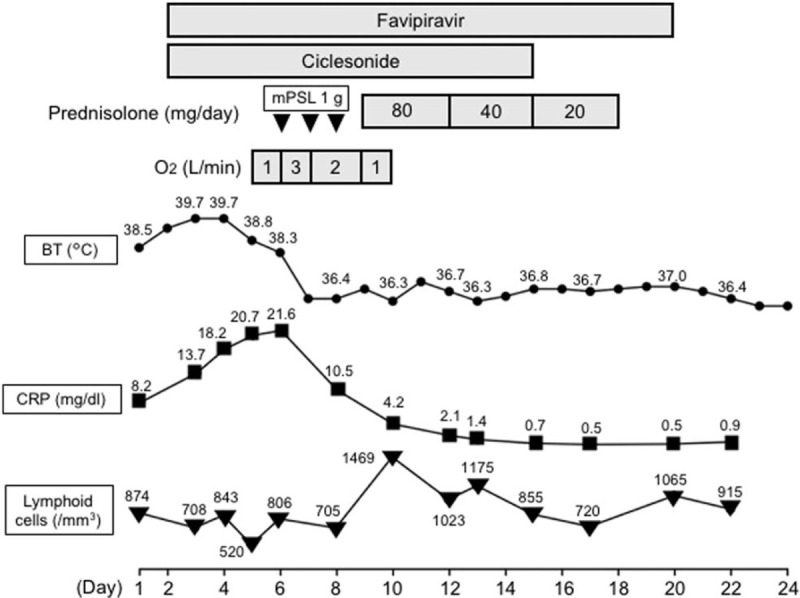
Clinical course of Case 1. Systemic corticosteroid treatment was initiated on day 6 post hospital admission. Subsequently, the patient's fever, respiratory condition, and laboratory data improved. mPSL = methylprednisolone, BT = body temperature, CRP = C-reactive protein.

On day 6 post hospital admission, he continued to have a fever and required 3 L/min oxygen via nasal canula to maintain an oxygen saturation of >90%. Serum CRP was 21.64 mg/dL and LDH was 541 U/L. Lymphopenia (806 cells/μL) and elevated serum ferritin (2,110 ng/mL) were observed. The patient had no signs of connective tissue disease (CTD), such as joint pain/swelling, proximal muscle weakness, and cutaneous rash. Serum creatinine kinase was not elevated, and anti-nuclear antibody tests were repeatedly negative. His chest X-ray revealed lung consolidation and marked lung volume reduction (Fig. 1 D). Further, his chest CT scan revealed consolidation, accompanied with reversed halo sign, traction bronchiectasis, and volume loss of the lower lobes (Fig. 1E, F). The patient was administered 1,000 mg methylprednisolone daily for 3 days, followed by a daily dose of 80 mg/day (1 mg/kg) prednisolone. His fever and chest X-ray improved rapidly, and his respiratory condition also improved (Fig. 2). Viral RT-PCR results were negative on day 16. Prednisolone was tapered without disease recurrence and the treatment ended on day 18. Chest imaging on day 23 revealed lung volume and infiltrate improvement (Fig. 1 G–I), and the patient was discharged on day 24.

### 2.2 Case 2

**Figure 3:**
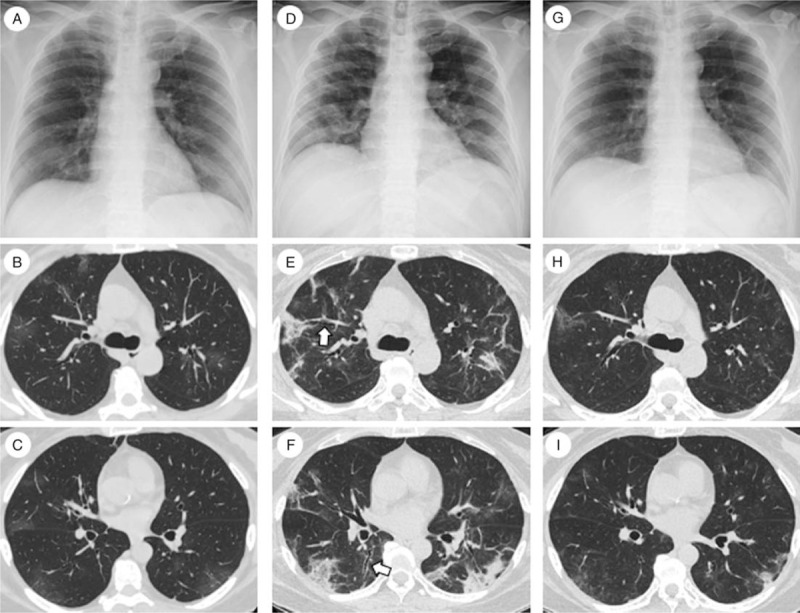
Chest imaging of Case 2. (A) Chest X-ray on admission. A slight ground-glass opacity (GGO) was observed in the right lower lung field. (B, C) Chest computed tomography (CT) on admission showed bilateral faint GGO. (D) Chest X-ray on day 6 post hospital admission (immediately before corticosteroid administration) showed bilateral GGO. Lung volume was markedly decreased. E, F: Chest CT on day 6. Consolidations developed bilaterally, some of which producing band-like shapes, and were distributed in subpleural or peri-bronchial regions. Traction bronchiectasis was observed (white arrow). A (G) Chest X-ray and (H, I) chest CT on day 17 revealed improvement of lung infiltrates and the recovery of lung volume. CT = computed tomography.

A 61-year-old woman presented with a 7-day history of fever, headache, and dysgeusia. She had a medical history of bronchial asthma, hypothyroidism, and hypertension. On admission, she had a pulse of 97 bpm, temperature of 38.4 °C, blood pressure of 141/91 mmHg, and oxygen saturation of 96% when breathing air. Laboratory data revealed lymphopenia (510 cells/μL) and elevated serum CRP (9.93 mg/dL) and ferritin (269 ng/mL) levels. Chest X-ray and CT showed slight GGO (Fig. 3 A–C). She was diagnosed with COVID-19 after receiving a positive test result via real-time RT-PCR for SARS-CoV-2. She was treated with ceftriaxone, azithromycin, favipiravir and hydroxychloroquine.

**Figure 4:**
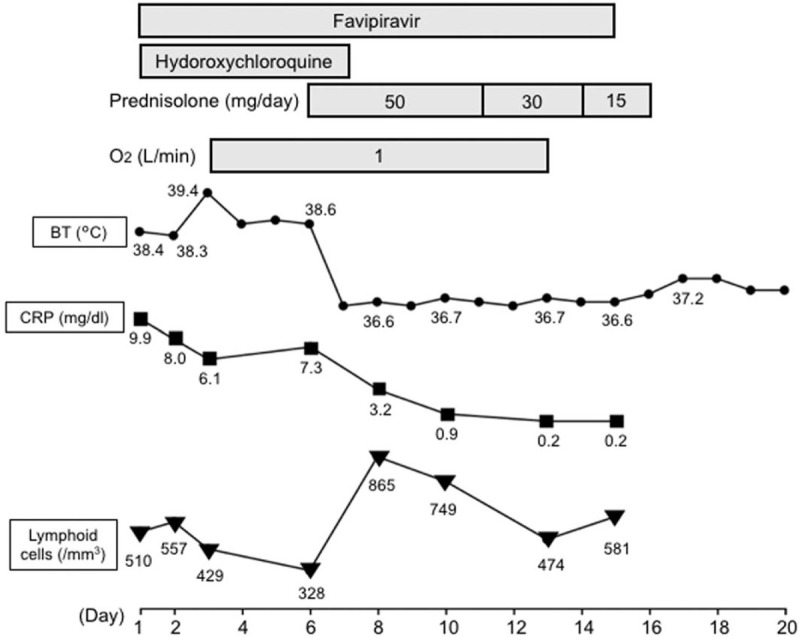
Clinical Course of Case 2. After the systemic corticosteroid treatment was initiated on day 6 post hospital admission, the patient's fever, respiratory condition, and laboratory data improved. BT = body temperature, CRP = C-reactive protein.

On day 6 post hospital admission, the patient's fever continued, she developed dyspnea and fatigue, and required 1 L/min oxygen via the nasal canula to maintain >90% oxygen saturation. Serum levels of CRP remained high (7.32 mg/dL), and lymphopenia (328/μL) and serum ferritin level (414 ng/mL) worsened. She had no signs of CTD. Serum creatinine kinase was not elevated, and both anti-aminoacyl-tRNA synthetase and an anti-melanoma differentiation associated gene 5 antibodies were negative. A chest X-ray revealed bilateral GGO and marked lung volume reduction (Fig. 3D). CT scan revealed bilateral consolidations, some of which showed band-like shapes and distributed in subpleural or peri-bronchial region. Traction bronchiectasis were also observed (Fig. 3E, F). We performed real-time RT-PCR for SARS-CoV-2 again and found an increased Ct value, which suggested decreased viral load. She was administered prednisolone 50 mg/day (0.75 mg/kg). Her fever and the level of serum CRP improved rapidly, followed by an improvement in the respiratory condition (Fig. 4). Chest imaging on day 17 revealed infiltrate and lung volume improvements (Fig. 3G–I), and a viral real-time RT-PCR test performed on the same day was negative. Prednisolone was tapered until the treatment concluded on day 18 without disease recurrence, and the patient was discharged on day 20.

## 3 Discussion

Here, we present two cases of rapidly progressive COVID-19 pneumonia. Chest imaging revealed the development of OP with fibrotic features and reduced lung volume. Notably, these findings improved with systemic corticosteroid treatment.

Several previous studies reported the development of OP during the clinical course of COVID-19.^[3–5]^ Copin et al confirmed the OP findings using autopsy lungs of patients who died from COVID-19.^[6]^ Typically, patients with OP display subacute disease over several weeks without apparent lung fibrosis.^[7]^ However, some patients with OP present with rapid disease progression and lung fibrosis, which is called “fibrosing variant of OP”.^[8–10]^ This degree of rapidly progressive and fibrotic OP is commonly observed in poly/dermatomyositis, especially in patients presenting with clinically amyopathic dermatomyositis associated with the anti- melanoma differentiation associated gene 5 antibody.^[11]^ However, neither of the case presented showed symptoms, physical findings, or serum antibodies suggestive of CTD, including myositis. Hence, our case report suggests that COVID-19 causes rapidly progressive and fibrotic OP.

The benefits and drawbacks associated with systemic corticosteroid treatment for COVID-19 are a subject of debate.^[12–14]^ Corticosteroids may control the “cytokine storm” frequently observed in COVID-19 patients,^[15,16]^ and a retrospective cohort study reported that treatment with methylprednisolone decreased the risk of death among COVID-19 patients with acute respiratory distress syndrome (HR 0.38, 95% CI 0.20-0.72).^[17]^ However, previous studies of viral infection, such as SARS, Middle East Respiratory Syndrome and Influenza virus pneumonia reported that systemic corticosteroid treatment was associated with unfavorable outcomes such as delayed virus clearance, increased incidence of hospital-acquired pneumonia, and increased mortality.^[18–20]^ Likewise, recent systematic review and meta-analysis showed that the use of corticosteroids may increase mortality of severe COVID-19 patients (HR 2.30, 95% CI 1.00 to 5.29) although the calculated effect size was based on only two observational studies and thus the quality of evidence was low.^[21]^ Several investigators have argued that that the timing of administration, and dosage of corticosteroid are important to adequately control the cytokine storm and prevent disease progression in COVID-19 patients.^[16,22]^ In our cases, we repeatedly performed real-time RT-PCR for SARS-CoV-2 and found that viral load was decreased when OP findings appeared. Therefore, we administered corticosteroid to prevent further deterioration of respiratory failure, and patients improved rapidly without any unfavorable effects.

Our report has several limitations. First, this is only a report of a small number of cases at 1 institution. It is still unclear whether these rapidly progressive and fibrotic cases are subtypes of COVID-19-associated OP. Further cases with similar presentations are needed. Second, although the 2 cases presented were successfully treated with corticosteroid therapy, it does not prove the efficacy of corticosteroid against COVID-19. Until the results of ongoing randomized controlled trials are available,^[23,24]^ we should carefully examine the indication of systemic corticosteroid according to each patient's situation.

## 4 Conclusion

Here, we reported 2 cases of COVID-19-associated OP, which presented with rapidly progressive respiratory failure and fibrotic changes on chest CT scan. For both cases, treatment with systemic corticosteroid rapidly resolved respiratory distress, and reversed lung fibrotic changes. Our findings suggest that physicians should carefully monitor patients with COVID-19 to prevent disease progression. Further investigation is needed to clarify the clinical characteristics of COVID-19-associated OP and confirm the potential benefit of corticosteroid treatment in COVID-19.

## Acknowledgments

We would like to thank all the members of the Keio COVID-19 Lifesaving Team, the Keio Donner Project Team, and the staff who supported us at the Keio University Hospital.

## Author contributions

**Conceptualization:** Satoshi Okamori, Hiroki Kabata.

**Diagnosis and treatment:** Ho Lee, Yasushi Kondo, Yuto Akiyama, Hiroki Kabata.

**Writing – original draft:** Satoshi Okamori, Ho Lee.

**Writing – review and editing:** Hiroki Kabata, Yuko Kaneko, Makoto Ishii, Naoki Hasegawa, Koichi Fukunaga.
